# Protocol for the economic evaluation of the diarrhea alleviation through zinc and oral rehydration salt therapy at scale through private and public providers in rural Gujarat and Uttar Pradesh, India

**DOI:** 10.1186/s13012-014-0164-2

**Published:** 2014-11-19

**Authors:** Samuel D Shillcutt, Amnesty E LeFevre, Christa L Fischer Walker, Robert E Black, Sarmila Mazumder

**Affiliations:** Department of International Health, Johns Hopkins Bloomberg School of Public Health, 615 N. Wolfe Street, Baltimore, 21205 MD USA; Centre for Health Research and Development, Society for Applied Studies, 45 KaluSarai, New Delhi, 110016 India

**Keywords:** Diarrhea, Zinc, Cost-effectiveness, Implementation science, India, Private sector, Community health

## Abstract

**Background:**

Child diarrhea persists as a leading public health problem in India despite evidence supporting zinc and low osmolarity oral rehydration salts as effective treatments. Across 2 years in 2010–2013, the Diarrhea Alleviation using Zinc and Oral Rehydration Salts Therapy (DAZT) program was implemented to operationalize delivery of these interventions at scale through private and public sector providers in rural Gujarat and Uttar Pradesh, India.

**Methods/Design:**

This study evaluates the cost-effectiveness of DAZT program activities relative to status quo conditions existing before the study, comparing a Monte Carlo simulation method with net-benefit regression, discussing the strengths and weaknesses of each approach. A control group was not included in the ‘before and after’ study design as zinc has proven effectiveness for diarrhea treatment. Costs will be calculated using a societal perspective including program implementation and household out-of-pocket payments for care seeking, as well as estimates of wages lost. Outcomes will be measured in terms of episodes averted in net-benefit regression and in terms of the years of life lost component of disability-adjusted life years in the method based on Monte Carlo simulation. The Lives Saved Tool will be used to model anticipated changes in mortality over time and deaths averted based on incremental changes in coverage of oral rehydration salts and zinc. Data will derive from cross-sectional surveys at the start, midpoint, and endpoint of the program. In addition, Lives Saved Tool (LiST) projections will be used to define the reference case value for the ceiling ratio in terms of natural units.

**Discussion:**

This study will be useful both in its application to an economic evaluation of a public health program in its implementation phase but also in its comparison of two methodological approaches to cost-effectiveness analysis. Both policy recommendations and methodological lessons learned will be discussed, recognizing the limitations in drawing strong policy conclusions due to the uncontrolled study design. It is expected that this protocol will be useful to researchers planning what method to use for the evaluation of similar before and after studies.

**Electronic supplementary material:**

The online version of this article (doi:10.1186/s13012-014-0164-2) contains supplementary material, which is available to authorized users.

## Background

Worldwide, diarrhea is the fourth leading cause of mortality among children under 5, accounting for 9% of total deaths [1]. In 2011, over 700,000 children died due to diarrhea [2], with eighty percent of cases occurring in East Asia and the Pacific, South Asia, and Africa, and 33% in South Asia alone [3]. However, diarrhea mortality is a solvable health problem, with this number of deaths having fallen from 4.6 million in 1980 [4]. India is a priority area for addressing the remaining burden, recognized as one of 15 countries that account for 53% of total episodes worldwide, with 312.22 million episodes and 205,600 deaths each year nationwide [2].

Since becoming widely used for diarrhea treatment in the 1980s [5], oral rehydration salts (ORS) have been instrumental in contributing to declines in prevalence. ORS prevents mortality by reducing the loss of fluids and electrolytes and death due to dehydration [6]. However, coverage of ORS in India remains low at less than 30%, and one out of ten children nationwide continues to experience diarrhea in any 2-week period [7].

The 2004 United Nations Children’s Fund (UNICEF)/World Health Organization (WHO) Joint Statement for the Clinical Management of Acute Diarrhea revised the global standards for acute diarrhea management to include ‘20 mg per day of zinc supplementation for 10–14 days (10 mg per day for infants under 6 months old)’ [8]. The therapeutic effect of zinc is to strengthen the immune system, improve absorption of water and electrolytes in the intestines, enhance the regeneration of the gut epithelium, increase levels of enzymes in the epithelium, and help the body clear pathogens from the intestines [9]. As a complement to ORS, zinc has been shown to reduce incidence [8], prevalence [10], and duration of diarrhea episodes [8],[9],[11]-[13]. Evidence on whether zinc reduces all-cause mortality is scarce, with one trial using non-injury mortality as a proxy for diarrhea deaths [9], not testing mortality as an outcome [11], or not having sufficient power to detect a significant difference in mortality [13]. Baqui et al. (2002) showed a non-significant difference in non-injury deaths, controlling for other factors, and authors concluded that the effect on mortality was due to zinc [9]. Applying the Child Health Epidemiology Reference Group (CHERG) Rules for Evidence Review indicates that mortality reduction could be as much as 23%, and ideal data, from randomized controlled trials (RCTs), is unlikely to emerge as the strength of evidence in support of zinc makes these trials unethical [14].

In India, evidence on the effectiveness of zinc for the management of acute diarrhea is mixed between studies showing an effect [10],[15],[16], and those with no or marginal effects [17]-[19]. Of these, one was conducted in a rural community setting [10]. This randomized controlled trial was carried out in six primary health care (approximately 30,000 population each centers) in Haryana [10], which provided an intervention that included training and supply of zinc and ORS to Anganwadi Workers and primary health center staff. This intervention nearly doubled the proportion of patients that received treatment between 3 and 6 months. In addition, the intervention led to significant reductions in diarrhea prevalence and the rate of hospitalizations due to diarrhea.

Building upon the success of effectiveness trial activities in Haryana, programmatic efforts to introduce zinc and ORS have been initiated throughout the last decade in India. Between 2005–10, the Point-of-Use Water Disinfection and Zinc Treatment (POUZN) project was implemented and found to be effective in improving both supply and demand for zinc. This program worked with pharmaceutical companies, channeled zinc through both the public and private sectors, collaborated with non-governmental organizations (NGOs), recruited support from key opinion leaders, provided training to detailers, stimulated prescribing practices among rural medical providers (RMPs), and promoted products with social marketing.

In 2010, the Diarrhea Alleviation using Zinc and ORS Therapy (DAZT) program expanded upon POUZN activities in scale and scope to make zinc and ORS available for the management of diarrhea through private and public sector providers in 6 districts of Gujarat and 12 districts of Uttar Pradesh (UP) states of northern India. DAZT is similar to POUZN in the characteristics mentioned above, but differed in that DAZT did not provide point-of-use water interventions, monitored zinc purchase and sales with short messaging service (SMS) in the private sector, and promoted zinc through informational booths in private clinics and hospitals. A full description of DAZT program activities is presented in Table 1.

**Table 1:** **Diarrhea Alleviation through Zinc and Oral Rehydration Therapy (DAZT) Program Summary**

Sectors	Program activities
Public sector	Micronutrient Initiative (MI)
State-level policy changes	• Permission to implement DAZT was formalized through Memorandums of Cooperation between MI and the state government, and MI and the Department of Health and Family Welfare of Gujarat
	• In Uttar Pradesh, less formal permission was obtained from the government
	• Commitment from the Department of Women and Child Development in Gujarat
	• Both states added zinc to their NRHM guidelines and essential drug lists
Programmatic planning	National Rural Health Mission (NRHM) Program Implementation Plans (PIPs) were changed to include the procurement of zinc and ORS
Training	Three levels of training were conducted including (1) district level supervisors, (2) Block level supervisors and health workers, and (3) ASHAs and AWWs. Trios, a Delhi-based agency, conducted training in Gujarat, and three NGOs conducted the training in UP
Supply	• Supply was provided by two pharmaceutical companies including Healthy Life Pharma and FDC limited assuming that the public sector would treat 10% to 15% of diarrhea cases
	• Kits contained two ORS sachets and 14 taste masked zinc tablets, a measuring cup, and an informational leaflet for caregivers
Procurement	• Healthy Life Pharma and FDC limited provided the first procurement of kits
	• In Gujarat, in phase 1 (2011), MI provided ORS and zinc and in phase 2 (2012), MI limited its provision to zinc only (government procured ORS)
	• In 2013, the state governments disbursed funds to all districts to purchase zinc
	• ANMs may have used supply procured from sources other than MI
Incentives	Incentives were delivered to ASHAs, AWWs, and ANMs at monthly meetings to increase attendance rates
Distribution	• Supplies were distributed from Healthy Life Pharma to district medical stores, to district hospitals or block offices/CHC/PHC, to HSC-ANMs and CDPOs, to ASHAs and AWWs
	• ANMs informed PHC block level supervisors about needs; supplies were redistributed from areas of surplus to areas of shortage
Monitoring and supervision	• Supportive supervisors and MI divisional coordinators provided supportive supervision at the district, block, sub-center, and village levels in the form of data validation and capacity building
	• These mechanisms complemented existing monitoring mechanisms of the public health system
	• Supervisors attended monthly meetings of ASHAs, AWWs, ANMs, spent at least 18 days monitoring field staff visits, provided staff with hands on training when necessary, analyzed service provider knowledge and skills, stock status, and caregiver compliance with treatment
**Private sector**	**Family Health International-360 (FHI-360)**
Policy changes	• Memorandums of understanding were signed with prominent professional medical organizations (IAP, IMA, and other local medical associations)
	• Partnered with NGOs, pharmaceutical companies, and homeopathic and alternative medicine associations
Programmatic planning	An implementation plan was developed which involved a push and pull strategy—push: changed prescription among key opinion leaders in the medical community and created IEC materials with medical experts about diarrhea management and marketed ORS and zinc to RMPs and drug sellers; pull: natural demand creation for ORS and zinc within this group
Training	• NGO and pharmaceutical staff trained for three days in diarrhea epidemiology, importance of zinc and ORS, correct dosage and regulatory guidelines, and promotional strategies for effective product placement
	• Professional organization were provided with continuing medical education
	• DAZT corner staff were trained on selected topics from the three day training schedule
	• In UP, ten RMPs from the Sehat Mitra project were trained with an adapted version of the three day training session
Supply	Local manufacturers were linked with informal providers in designated areas
Procurement	Utopia Pharmaceuticals and Prayas manufactured and distributed zinc in UP, and RMPs procured zinc from West Coast Pharmaceuticals and generic brands from NGOs in Gujarat, with procurement plans accounting for different levels of demand according to season
Incentives	Pharma companies provided field representatives with commissions of 2 Rupees for each sale above 200
Distribution	Generic distributors supplied District Coordinator offices, which distributed to the Tehsil Coordinator based on demand
DAZT corners	Staffed informational booths in private clinics and hospitals to create awareness among caregivers and remind providers to prescribe zinc
Sehat Mitra project	In Faizabad Uttar Pradesh, a pilot project to provide ORS and zinc in patient’s homes by RMPs traveling on bicycles
Monitoring and supervision	• Monthly NGO and pharma staff meetings, validation of data and reports, SMS messaging from the field
	• FHI staff attended monthly meetings, district coordinators spent a lot of time in the field working with new staff

Accredited Social Health Activists (ASHAs); Anganwadi Workers (AWWs); Auxiliary nurse midwives (ANM); Child development project officer (CDPO); Community health centers (CHC); Diarrhea Alleviation and Zinc Therapy (DAZT); FDC Limited pharmaceutical company (FDC); Health subcenter (HSC); Indian Academy of Pediatrics (IAP); Indian Medical Association (IMA); Information, education, communication (IEC); Micronutrient Initiative (MI); Non-governmental organizations (NGO); National Rural Health Mission (NRHM); Oral rehydration salts (ORS); Primary health centers (PHC); Program Implementation Plans (PIP); Rural medical providers (RMP); Short messaging service (SMS); Uttar Pradesh (UP).

The objective of this study is to evaluate the cost-effectiveness of the DAZT program under real-world conditions compared to the status quo existing before the intervention was introduced in the study area. Two analytical approaches will be used to meet this objective: calculation of cost-effectiveness using a Monte Carlo Simulation method with patient level data [20], and calculation of cost-effectiveness using a net-benefit regression approach to control for covariates [21].

Both community-based [Lefevre et al. forthcoming, Bishai et al. forthcoming] and model-based evidence [4],[22] suggest that zinc supplementation to treat diarrhea is cost-effective in low- and middle-income countries (LMICs), although hospital-based studies do not find a significant difference in cost [23], effect [19], or both [24] between children receiving zinc and those that do not. These studies were conducted across a wide range of settings; however, none of them can be classified as implementation research. This study will evaluate the cost-effectiveness of the DAZT program and further discourse on approaches to economic evaluation of health programs in low- and middle-income countries. Methodologically, examples of cost-effectiveness analyses evaluating ‘before and after’ studies designs are emerging (for example [25],[26]); however, these types of studies are not well established in the literature. Thought is needed to identify special considerations that may be relevant to before and after study evaluations. The International Society for Pharmacoeconomics and Outcomes Research (ISPOR) has developed guidelines for using ‘real-world data’ [27], but guidelines for conducting cost-effectiveness analysis (CEA) alongside ‘before and after’ studies do not exist.

Alternative methods for conducting CEA are sometimes used to evaluate a single dataset to make methodological points, assess validity, and test robustness of results according to structural uncertainty [21],[28],[29]. Two of several analytical approaches available for CEA include Monte Carlo Simulation methods with patient level data [20] and net-benefit regression [21]. The latter framework combines established methodologies from economic evaluation and econometrics and has the advantage of being able to adjust for imbalances in confounding variables. Net-benefit regression methodology can be used for randomized [21],[30] and non-randomized studies [31],[32], although the case can be made that it is even more appropriate for non-randomized study designs as covariates are more likely to be unevenly distributed across study arms [33]. Bootstrapping confidence intervals presents the problem of ambiguity in incremental cost-effectiveness ratios (ICERs), although this problem can be circumvented in both methods using the net-benefit statistic, and both net-benefit regression and bootstrapping can be used to generate cost-effectiveness acceptability curves (CEACs). The bootstrap approach offers one advantage in that uncertainty around program costs can be simulated by resampling from parametric distributions fit to the data, while program costs are divided evenly across patients within each arm in the net-benefit regression approach [21] or are assumed to cancel out across study arms or phases [31]. Deterministic calculations and net-benefit regression are commonly evaluated in tandem in the literature [21],[31],[32], but scope exists to add thought on comparisons between bootstrapping and net-benefit regression. It is anticipated that documenting thought behind choosing between these analytic frameworks will be useful to researchers facing similar questions about evaluation of programs with similar study designs.

Policy recommendations from the completed research will add to the growing literature evaluating the cost-effectiveness of zinc for diarrhea treatment in low- and middle-income countries. Findings will be timely as India is developing a Health Technology Assessment (HTA) program in collaboration with the UK National Institute of Health and Care Excellence [34], in which the importance of CEA in policy making can be expected to increase. In addition, local capacity to produce zinc is rapidly expanding including Bharat Immunologicals and Biologicals Corporation Limited (BIBCOL), which is a government-owned corporation with the capacity to produce 240 million of 20 mg scored tablets of zinc sulfate per year [35]. Currently, about 40 private companies are producing zinc for local and international markets.

This article presents the DAZT program in India. Analytic methods for evaluating cost-effectiveness are described and compared, in addition to methods for assessing wealth quintile using principal components analysis. Strengths and limitations of the study are discussed, followed by arguments against targeting zinc according to subgroup, and considerations necessary for drawing conclusions about generalizability.

## Methods

### Setting and study design

Gujarat and Uttar Pradesh are states in northwest and north India, respectively, which, along with Madhya Pradesh, contribute to half of the diarrhea burden in India. The majority of these states are rural, access to basic sanitation is limited, and most adults have only primary education or less; although signs exist that these states are progressing through the ‘demographic transition’ [36],[37]. In 2010, the DAZT program was introduced through both the public and private sectors in 6 districts of Gujarat and 12 districts of Uttar Pradesh. Care seeking for diarrhea was mostly from private doctors, and shifts in use of the health system were seen from private hospitals to community health workers. Data were collected on the last episode affecting the youngest child in each household surveyed Given that the intervention is implemented on a system-wide level, and the effectiveness of zinc has been established for diarrhea treatment [14], a ‘before and after’ study design was chosen [38]. This evaluation compares the costs and health outcomes associated with the DAZT intervention after 2 years of implementation (Table 1) with those that preceded the intervention. This time frame was considered appropriate as it represented conditions after which the program was fully implemented. Outcome indicators include caregiver knowledge, incidence of diarrhea, treatment seeking, source of care, use of zinc, cost of treatment, and cost-effectiveness.

### Sample size and power calculation

The sample size for the main effects study was calculated according to standard methods for rates calculated through cluster randomized designs. Calculations used ORS coverage rates in the initial survey since very few children received zinc before the start of the program, and delivery of ORS and zinc were assumed to be linked. Actual sample sizes achieved for each survey are presented in Table 2. Using sample sizes from the main study, power to detect meaningful differences will be calculated for our cost-effectiveness estimate. Two formulas from Glick et al. [39] will be merged to determine power for cost-effectiveness using a novel approach to calculating the reference case ceiling ratio from an estimate based on per capita gross national income (GNI) and Lives Saved Tool (LiST) outputs of deaths averted/episodes averted.

**Table 2:** **Sample sizes for each survey**

Survey	Number of participants		Dates
	Gujarat	Uttar Pradesh	
Starting point	4,200	3,889	March 22–May 10, 2011 Gujarat; April 1–June 21, 2011 Uttar Pradesh
Monsoon season			June through beginning of September^a^
Midpoint	1,072	1,790	September 1–October 8, 2012 Gujarat; May 24-October 4 2012 Uttar Pradesh
Endpoint	5,080	1,001	September 28–November 18, 2013 Gujarat; August 25–October 12, 2014 Uttar Pradesh

^a^Although peak diarrhea season lasts until November.

### Sampling and data collection

Data were collected through closed-ended survey questionnaires administered to a sample of caregivers of children 2–59 months old. In each survey, information was elicited on caregiver’s knowledge of diarrhea management, illnesses in the past 2 weeks, care seeking, treatment received, wealth quintile, and demographic characteristics of the household. Data on economic costs will be collected prospectively throughout implementation to capture program and incremental users' costs.

Program costs will be based on the financial records of the international NGOs working with public and private sector partners. To enhance generalizability, capital costs of furniture and equipment will be based on the original price paid inflated to 2014 and annualized using the full lifespan of the item and a 3% discount rate [20]. Donated labor, equipment, and facilities will be valued according to market prices; transfer payments such as taxes and subsidies will be removed; and rent costs will be used to represent building costs [40]. Since results will be presented in US$, it will not be necessary to adjust non-traded items to correct for price distortions [41]. Users' cost will be derived from reported out-of-pocket payments for care seeking and wages lost.

### Conceptual framework

**Figure 1:**
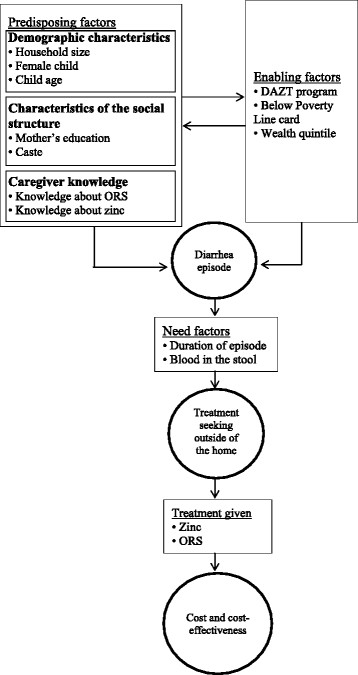
**Conceptual framework based on Andersen and Newman [**[42]**].**

A conceptual framework has been developed to identify important model variables based on categories from Andersen and Newman [42]. This framework consists of three main categories of interacting factors that are associated with treatment seeking including predisposing, enabling, and need factors; as indicated in Figure 1. The model was limited to only children that sought care for diarrhea, although other symptoms associated with diarrhea were assessed in caregiver surveys. If treatment was sought and given, economic costs could result, which are an important component of cost-effectiveness. In our framework, knowledge differs from the concept described in Andersen and Newman [43] in that our data describes awareness of the treatments, where they consider values and beliefs about treatment. All of these variables were justified by literature, and will be included regardless of whether their coefficients in our models are significant to ensure that variances are correctly estimated.

### Descriptive statistics

Descriptive statistics of the parameters (Table 3) will be calculated according to their means, confidence intervals, and *p*-values reflecting the extent of difference between arms. Comparisons will be made at the start and endpoint of the program. Stata svy commands will be used to account for clustering with *F*-tests used to evaluate the significance of differences between groups. As costs are not normally distributed, significance of differences between components listed in Table 4 at the start and end of the program will be calculated using confidence intervals with bootstrapped standard errors.

**Table 3:** **Variables to be tested**

Conceptual framework category	Parameter	Description
Demographic characteristics	Household size	Continuous variable (min = 2 people, max = 23 people in Gujarat and 32 in Uttar Pradesh)
	Child sex	Male =0, female =1
	Child age	Continuous variable (min =2 months, max =59 months)
Characteristics of the social structure	Mother’s education	No or primary education =0, primary education or above =1, secondary education or above =2, tertiary education or above =3
	Father’s education	No or primary education =0, primary education or above =1, secondary education or above =2, tertiary education or above =3
	Scheduled caste	Not a scheduled caste =0, scheduled caste =1
	Scheduled tribe	Not a scheduled tribe =0, scheduled tribe =1
	Other backwards caste	Not another backwards caste =0, other backwards caste =1
Caregiver knowledge	Knowledge about ORS	No knowledge =0, knowledge =1
	Knowledge about zinc	No knowledge =0, knowledge =1
Enabling factors	DAZT program	Initial survey, final survey
	Below poverty line card	No BPL card =0, BPL card =1
	Very poor	Any other wealth quintile =0, second wealth quintile =1
	Poor	Any other wealth quintile =0, third wealth quintile =1
	Less poor	Any other wealth quintile =0, fourth wealth quintile =1
	Least poor	Any other wealth quintile =0, fifth wealth quintile =1
Need factors	Duration of diarrhea	Continuous variable (min = 0 days, max = 15 days in Gujarat and 32 in Uttar Pradesh)
	Blood in the stool	No blood in the stool =0, Blood in the stool =1
Treatment seeking	Seek treatment outside of the home	No treatment sought outside of the home =0, treatment sought =1
Treatment given	Given ORS	No ORS =0, given ORS =1
	Given zinc	No zinc =0, given zinc =1

**Table 4:** **Descriptive statistics about costs**

	Costs according to source of care and components according to outpatient, inpatient, and home care
Costs according to source of care	
Public source—facility care	PHC, government hospital, government dispensary
	Auxiliary nurse midwife, sub-center
Public source—community care	Anganwadi worker/center
	ASHA
Private source	Private doctor
	Nursing home/private hospital
	Mobile clinic
	Chemist
	Traditional healer
	Charitable hospital, NGO, trust
Cost components according to outpatient, inpatient, and home care	
Direct medical	Consultation
	Dispensing
	Purchase of zinc (tablets or syrup)
	Purchase of ORS (packets)
	Purchase of other drugs
	Special food purchased
	Admission/hospitalization
	Other costs
Direct nonmedical	Transportation (round trip)
Indirect costs	Wages lost

ASHA: Accredited Social Health Activist; NGO: Non-governmental organization; ORS: Oral rehydration salts; PHC: Primary health center.

### Costing

The cost of the DAZT program from 2011 to 2013 (Gujarat) and 2014 (Uttar Pradesh) will be calculated from the societal perspective according to Saving Newborn Lives guidelines and standard textbooks [20],[40],[43]. The societal perspective reflects the incremental costs incurred by implementing agencies, government providers, and households. Program costs will be divided into capital costs and recurrent costs, with data derived from program records, primary sources, and interviews with individuals responsible for implementation. Capital costs will be annualized and discounted according to a rate of 3% according to the standard rate for economic evaluation in International Health [44]. Consumer price indices from the International Monetary Fund (IMF) will be used to inflate costs to 2014. Costs will be converted to US$ using exchange rates from OANDA.com. Incremental government provider costs will be derived using reported estimates of time spent on the provision of diarrhea treatment services by public sector providers. Economic costs to caregivers for diarrhea treatment of the episodes in the last 2 weeks will be evaluated according to data collected through household questionnaire and consist of direct medical costs of treatment, direct non-medical costs (transportation), and indirect costs (wages lost by caregivers) [45]. No adjustments will be made to collected data to approximate opportunity costs.

### Health outcomes

In the standard cost-effectiveness calculation, health outcomes will be derived from incremental changes in coverage measured in cross-sectional household surveys and inputted into LiST. LiST will estimate the incremental number of lives saved from the study’s start to its completion. The years of life lost (YLL) component of disability-adjusted life years (DALYs) will be calculated using the standard formula from the Global Burden of Disease study [46]. A life expectancy estimate will be taken from WHO life tables representing the midpoint between 1–4 years old. A discount rate of 3% will be used in reference case calculations, and age weighting will not be used to be consistent with the Disease Control Priorities Project second edition (DCP2) [41]. Long-term sequelae such as acute cognitive and psychomotor development effects in young children, the effects of diarrhea on stunting [4], and obesity and related conditions that can occur later in life (cardiovascular disease and diabetes) [47] were not measured and will not be considered. The net-benefit regression approach will be limited to episodes averted as it is impossible to know the specific long-term health outcomes for individual patients. Limitations of drawing data from a single uncontrolled study are recognized [48], although cost-effectiveness analysis is often conducted on data from a single effectiveness study, and conditions in the study sites are not atypical of other relevant settings in low- and middle-income countries.

### Cost-effectiveness analysis

Cost-effectiveness will be calculated using two different approaches including 1) Monte Carlo Simulation with patient level data [20] and 2) a net-benefit regression-based approach [21].

### Monte Carlo Simulation with patient level data approach to cost-effectiveness analysis

Initial cost-effectiveness calculations will be generated through Monte Carlo simulation to evaluate uncertainty in economic costs to caregivers and health outcomes, using an additional simulation technique to account for the uncertainty in program costs. Samples of economic costs to caregivers and health outcomes will be drawn from the data equal in size to the number of data points, with replacement. Uncertainty around program costs will be simulated by drawing data from gamma distributions derived from most likely, low and high estimates for each component. This process will be repeated 10,000 times to ensure that tails of standard error distributions are filled and increase stability in results since available computing power is sufficient. ICERs will be calculated, and uncertainty will be quantified using a bootstrap approach, choosing the values representing the upper and lower 2.5% of data. To disambiguate negative ICERs and show the probability of cost-effectiveness according to different values of the ceiling ratio, CEACs will be calculated, using per capita GNI as the reference case threshold [49] (US$1,740 in Gujarat, $571 in Uttar Pradesh). Calculations will be performed using Microsoft Excel with the Palisade at-risk add-in.

### Net-benefit regression-based approach to cost-effectiveness analysis

A second approach to calculating cost-effectiveness will be taken according to net-benefit regression methods to control for imbalances in covariates arising from the non-randomized study design [21],[31],[32]. This method uses patient-level data of economic costs to caregivers and health outcomes, distributing program costs from the last year of the program evenly across children in the endpoint survey. A modification of the net-benefit statistic will serve as the dependent variable, given that outcomes are measured in terms of a health gap measure instead of health expectancy measure [50]. This formulation adds costs to outcomes expressed in monetary terms instead of taking the difference as is done in standard net-benefit calculations. The coefficient on a treatment variable that distinguishes between the starting point and endpoint of the program will be used to represent incremental net benefit. One sided *p*-values from the treatment variable will be used to construct CEACs, using results from a series of regressions that use different values for the ceiling ratio. Due to the non-normality in the distribution of residual values, a generalized linear model using a gamma distribution will be used. Huber-White robust standard errors will be used to address heteroskedasticity, and clustering will be accounted for consistent with study design. Data will be checked for non-linearities to assess whether use of a spline term improves model fit. The importance of influential data points will be checked using DFBETAs, excluding caregivers with the top ten DFBETA scores in sensitivity analysis. Calculations will be performed in Stata 13, using standards for good practice for using statistical regression models in economic evaluations [51].

### Principal components analysis

Principal components analysis will be used to categorize caregivers according to wealth quintile using established methods [52],[53]. While no established rules exist for selecting variables, broad categories of assets will include durable asset ownership, housing characteristics, and access to basic services based on precedent [52]. To avoid illogical rankings [54], only those variables with a prevalence between 5%–95% will be retained. While these thresholds are arbitrary, they are consistent with rules of general inference to define a value with low probability. Eigenvalues will be generated in Stata, which represent linear combinations of variables that capture the maximum amount of remaining uncertainty in the data for each component. The component with the greatest eigenvalue will be selected for creating a wealth index, as higher order components have been shown not to be important in a previous study and convention has become to create wealth indices on only one component [52]. Factor loadings will be assessed to determine what variables align the most closely with the principal component and evaluate whether its representation of wealth has face validity. Caregivers will be divided into quintiles, and variables for regression analyses will be based on these quintiles. The scale will be validated according to a Cronbach’s alpha greater than 0.6.

### Ethical approval and study status

For the main study, ethical approval was obtained from SAS and the Johns Hopkins Bloomberg School of Public Health (JHSPH) Institutional Review Board (IRB). DAZT project activities commenced in late 2010 and will span through 2014.

## Discussion

Economic evaluation provides insights into the appropriate allocation of resources, and yet the number of cost-effectiveness and cost-utility analyses alongside programs at scale - where large amounts of resources are invested - remains limited. When planning these studies, differences in methods adopted have implications on the validity of findings and on their generalizability. Economic evaluation has traditionally focused on the cost of an intervention to produce a desired health outcome in studies based on modeled evidence or data collected alongside randomized controlled trials. As the number of interventions with known impact on health increases, there is an increased need for evidence on ways of operationalizing delivery of these health services to ensure high coverage, quality, and cost-effectiveness.

The aims of the DAZT intervention are to improve the supply, demand, and prescribing practices for treatment of diarrhea in children with zinc and ORS. These objectives are consistent with goals of The Indian Academy of Pediatrics [55], WHO [56], and UNICEF [3] who have all endorsed zinc supplementation to ORS for the treatment of diarrhea in children. In addition, the Government of India has established the Oral Rehydration Therapy Program to increase awareness about causes of diarrhea and its treatment among mothers and communities [7], and results of this study may be used to support this program.

This study will provide an evaluation of a zinc delivery strategy in a resource-constrained setting, in addition to a comparison of methods for cost-effectiveness analysis. The calculation of results using two different methods will provide evidence about the robustness of results, while informing broader discourse on methods for carrying out economic evaluations alongside programs delivered at scale. Each method has its strengths and limitations. The Monte Carlo Simulation with patient level data approach will allow for the incorporation of uncertainty around program costs and is a more widely used technique than the net-benefit regression approach. The net-benefit regression framework will allow for cost-effectiveness to be calculated controlling for other factors, which are unbalanced across arms in this study. Comparing the two analytical approaches will stimulate discussion about when analysts should choose one over the other when planning their analyses, particularly in the context of evaluation of programs at scale.

### Strengths and limitations

The main strength of this study is that it provides two different analytic perspectives on cost-effectiveness, allowing assessment of the robustness of results. It’s time frame is comparable to other community-based studies, although the full effect may not be captured as knowledge takes time to fully proliferate, supply systems mature through time, and changes in prescribing patterns can be a gradual process. A heterogeneous mix of patients will be included, enhancing the ability to generalize findings to other areas in India. Recall bias is minimized by asking only about episodes occurring in the last 2 weeks, in keeping with precedent from a previous study [57]. Overall, the quality of the data is high, with interviewers returning to households to fill gaps in missing data.

The main limitation of this study is that the study design of uncontrolled before and after studies is vulnerable to bias, such as secular trends, the Hawthorne effect [48], or regression to the mean [58]. However, the case for clinical effectiveness can be made based on evidence from randomized studies that have found reductions in the prevalence of diarrhea when providers were enabled to give zinc to children with diarrhea [10]. A CHERG review concluded that zinc causes a 23% reduction in diarrhea-specific mortality based on reductions in hospitalization rates, reasoning that this estimate was realistic as it was more conservative than reductions in all-cause or diarrhea-specific mortality found in other studies [14]. This evidence is consistent with the 2004 WHO/UNICEF endorsement of zinc for treating child diarrhea, which concluded that zinc reduces the severity and duration of acute diarrhea, and the number of episodes in the 2–3 months after treatment [8]. Since equipoise existed for the DAZT delivery strategy, but not for the effectiveness of zinc, the before and after study design was deemed to be the appropriate form of evaluation [59]. The intervention has since been rolled out statewide in Gujarat, and the final survey is underway in Uttar Pradesh.

Secular trends such as improvements in living conditions, access to safe water, and improved sanitation may have contributed to the decline in diarrhea prevalence in the DAZT study area [60]. However, secular trends may be less relevant for costs as economic growth is unlikely to drive prices for diarrhea treatment down. In addition, this study design is sensitive to sudden changes in the conditions of the study area [61]. The Hawthorne effect is a potential confounder, which would overstate the magnitude of the effect [48]. Regression towards the mean may be problematic [58], which means that if a study had high diarrhea prevalence at beginning, future measurements will be likely to be less extreme. The DAZT study found a 14% prevalence of diarrhea in 2011, while levels were 13% in 2005/6 in Gujarat [36] and were 9% nationwide [7]. Regression to the mean predicts that the prevalence would be lower in future surveys as downward changes would be more frequent than upward changes.

Limitations exist in that household data is based on self-report, reporting bias could have occurred, and long-term outcomes and programmatic outcomes such as quality are not measured. Some researchers argue that only children who receive the recommended 10–14 day course should be included in analysis. However, in the DAZT program, most children only received partial courses of zinc in addition to antibiotics and anti-diarrheal medications. Seasonal effects are possible given that each survey was given at a different point in the monsoon cycle across the years of the study. Limitations of principal components analysis are recognized, such as not evaluating the quality of assets, assuming the first principal component to be an adequate indicator of socioeconomic status, being a relative measure, and producing quintiles with variable arrays of asset ownership [53],[62],[63]. Finally, outcomes will not be evaluated according to severity, which could lead to outcomes such as children with fewer symptoms or shorter episodes having more favorable cost-effectiveness.

### Targeting interventions according to subgroup

Cost-effectiveness according to subgroup can be assessed in the net-benefit regression framework as the coefficients on interaction terms between specific variables and the treatment variable. However, the case exists that zinc for diarrhea should not be targeted as it does not pose the threat of microbial resistance that is relevant to antibiotics and antimalarial medicines [64]. If widespread use of zinc reduces the use of antibiotics, microbial resistance to them may be slowed. In addition, zinc incurs only modest drug costs per child, and the costs of rationing treatment may outweigh the benefits of universal coverage [65]. In addition, universal coverage may be justified on equity grounds — while diarrhea affects people regardless of socioeconomic status, mortality preferentially affects the poor. Making treatments available according to patient subgroups is justified according to treatment effect, although is not justified according to demographic characteristics such as race [66]. However, information on key factors affecting effectiveness, such as or level of zinc status, were not collected. In addition, the data structure produces counterintuitive results as evaluating number of episodes treated at different service providers implies episodes averted when treatment seeking is reduced. For these reasons, cost-effectiveness according to subgroup will not be emphasized in this analysis.

### Generalizability

Results may be transferable to countries with similar health systems and epidemiological profiles. Because of differences in costs and similarities in efficacy of zinc across settings, the case has been made that cost-effectiveness is likely to vary more than effectiveness [22]. However, considerable heterogeneity in effectiveness has been found across settings, even within Asia [13], and results may be limited in their generalizability in this dimension as well. Other factors that may affect generalizability include ages of children, duration of illness, health systems infrastructure, receptiveness of health workers to changing treatment practices, and use of other medicines to treat diarrhea. Methodological assumptions may need adjustment as well, including the discount rate and study perspective. Descriptive statistics about the study setting will be presented to facilitate discussion about generalizability.

## Authors’ original submitted files for images

Below are the links to the authors’ original submitted files for images. Authors’ original file for figure 1 Authors’ original file for figure 2 Authors’ original file for figure 3 Authors’ original file for figure 4 Authors’ original file for figure 5

## Abbreviations

BMGF: Bill and Melinda Gates Foundation
CEA: cost-effectiveness analysis
CEAC: cost-effectiveness acceptability curves
CHAI: Clinton Health Access Initiative
DALY: disability-adjusted life years
DAZT: Diarrhea Alleviation using Zinc and ORS Therapy
DCP2: Disease Control Priorities Project second edition
FHI-360: Family Health International-360
GNI: gross national income
HTA: Health Technology Assessment
ICERs: incremental cost-effectiveness ratios
IMF: International Monetary Fund
IRB: Institutional Review Board
ISPOR: International Society for Pharmacoeconomics Research
JHSPH: Johns Hopkins Bloomberg School of Public Health
MI: micronutrients initiative
ORS: oral rehydration salts
PHCs: primary health centers
POUZN: Point-of-Use Water Disinfection and Zinc Treatment program
UNICEF: United Nations Children’s Fund
UP: Uttar Pradesh
WHO: World Health Organization
YLL: years of life lost
