# Supplementary figures and images for: Protocol for the economic evaluation of the diarrhea alleviation through zinc and oral rehydration salt therapy at scale through private and public providers in rural Gujarat and Uttar Pradesh, India
Source: Implement Sci. 2014 Nov 19;9:164. doi: 10.1186/s13012-014-0164-2 (PMC4335371; doi:10.1186/s13012-014-0164-2)

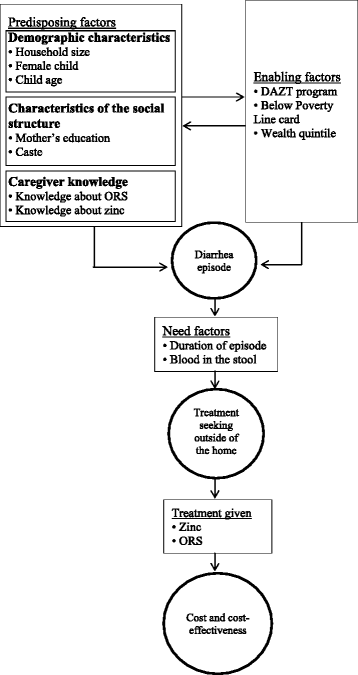

Supplement: Supplementary file 1 — Authors’ original file for figure 1 [file 13012_2014_164_MOESM1_ESM.gif]
