# Supplementary material for: Protocol for the economic evaluation of the diarrhea alleviation through zinc and oral rehydration salt therapy at scale through private and public providers in rural Gujarat and Uttar Pradesh, India
Source: Implement Sci. 2014 Nov 19;9:164. doi: 10.1186/s13012-014-0164-2 (PMC4335371; doi:10.1186/s13012-014-0164-2)
Supplement: Supplementary file 2 — Authors’ original file for figure 2 [file 13012_2014_164_MOESM2_ESM.docx]

**Table 1.** Diarrhea Alleviation through Zinc and oral rehydration Therapy (DAZT) intervention[[7](#_ENREF_7), [26](#_ENREF_26)]

| Public sector - Micronutrient Initiative (MI) |
| --- |
| Alliances: Between MI and the government. Commitment from the Department of Women and Child Development |
| Policy change: National Rural Health Mission (NRHM) Program Implementation Plans (PIPs) were changed to include the procurement of zinc and ORS |
| Training: MI trained three levels of supervisors, and supported the government in training public sector providers |
| Supply: Kits contained two ORS sachets and 14 taste masked zinc tablets, a measuring cup, and an informational leaflet for caregivers |
| Procurement: Healthy Life Pharma provided the first procurement of kits, phase 1 (2011) MI provided ORS and zinc, phase 2 (2012) MI limited to zinc only (government procured ORS), 2013 state disbursed funds to all districts to purchase zinc |
| Program implementation: Incentives delivered to ASHAs, AWWs, and ANMs at monthly meetings |
| Distribution: ANMs informed PHC block level supervisors about needs, supplies were redistributed from areas of surplus to areas of shortage |
| Monitoring and supervision: Each packet and referral was documented in a form, district coordinators routinely matched reports to data registers |
| Supportive supervision - Supervisors attended monthly meetings of ASHAs, AWWs, ANMs, spent at least 18 days monitoring field staff visits, provided staff with hands on training when necessary, analyzed service provider knowledge and skills, stock status, and caregiver compliance with treatment |
| Private sector - FHI-360 |
| Alliances: Memorandums of Understanding with prominent professional medical organizations (IAP, IMA). Partnered with four NGOs, West Coast Pharmaceutical Company, and homeopathic and alternative medicine associations |
| Policy change: None |
| Training: NGO and pharma field staff were trained in diarrhea epidemiology, importance of zinc and ORS, correct dosage and regulatory guidelines, promotional strategies, role of FHI-360, and use of an SMS MIS system. Continuing medical education was provided for professional organizations. Three day residential training was provided for NGO and pharmaceutical staff |
| Supply: Same as public sector |
| Procurement: All supplies procured through the public sector |
| Program implementation: Push and pull strategy – push: changed prescription among key opinion leaders in the medical community, pull: NGOs created IEC materials with medical experts about diarrhea management and marketed ORS and zinc to RMPs and drug sellers |
| DAZT corners: staffed informational booths to create awareness and remind providers to prescribe zinc |
| Sehat Mitra project: In Faizabad, in-home provision by RMPs on bicycles |
| Monitoring and supervision: Monitor activities, validate data and reports, SMS messaging from the field |
| Supportive supervision: FHI staff attended monthly meetings, district coordinators spent a lot of time in the field working with new staff |

Accredited Social Health Activists (ASHAs); Anganwadi Workers (AWWs); Non governmental organizations (NGO); Oral rehydration salts (ORS); Short messaging service (SMS); Rural medical providers (RMP); Indian Academy of Pediatrics (IAP); Indian Medical Association (IMA)
