# Supplementary material for: Protocol for the economic evaluation of the diarrhea alleviation through zinc and oral rehydration salt therapy at scale through private and public providers in rural Gujarat and Uttar Pradesh, India
Source: Implement Sci. 2014 Nov 19;9:164. doi: 10.1186/s13012-014-0164-2 (PMC4335371; doi:10.1186/s13012-014-0164-2)
Supplement: Supplementary file 3 — Authors’ original file for figure 3 [file 13012_2014_164_MOESM3_ESM.docx]

**Table 2.** Sample sizes for each survey

| Survey | Number of participants | | Dates |
| --- | --- | --- | --- |
|  | Gujarat | Uttar Pradesh |  |
| Starting point | 4,200 | 3,889 | March 22-May 21, 2011 |
| Monsoon season |  |  | June through beginning of September* |
| Midpoint | 1,072 | 1,790 | September 14-October 8, 2012 |
| Endpoint | 5,080 | forthcoming | October - December, 2013 |

*Although peak diarrhea season lasts until November
