# Supplementary material for: Protocol for the economic evaluation of the diarrhea alleviation through zinc and oral rehydration salt therapy at scale through private and public providers in rural Gujarat and Uttar Pradesh, India
Source: Implement Sci. 2014 Nov 19;9:164. doi: 10.1186/s13012-014-0164-2 (PMC4335371; doi:10.1186/s13012-014-0164-2)
Supplement: Supplementary file 4 — Authors’ original file for figure 4 [file 13012_2014_164_MOESM4_ESM.docx]

**Table 3.** Variables to be tested

| Parameter | Description |
| --- | --- |
| Study phase | Baseline, midline, endline |
| Household size | Continuous variable (min = 2 people, max = 23 people) |
| Child sex | Male = 0, female = 1 |
| Father primary education | No education = 0, Primary education or above = 1 |
| Father secondary education | No or primary education = 0, Secondary education or above = 1 |
| Mother primary education | No education = 0, Primary education or above = 1 |
| Mother secondary education | No or primary education = 0, Secondary education or above = 1 |
| Knowledge about ORS | No knowledge = 0, Knowledge = 1 |
| Knowledge about zinc | No knowledge = 0, Knowledge = 1 |
| Below poverty line card | No BPL card = 0, BPL card = 1 |
| Scheduled caste | Not a scheduled caste = 0, Scheduled caste = 1 |
| Scheduled tribe | Not a scheduled tribe = 0, Scheduled tribe = 1 |
| Other backwards caste | Not another backwards caste = 0, Other backwards caste = 1 |
| Duration of diarrhea | Continuous variable (min = 0 days, max = 15 days) |
| Private provider | Public provider = 0, Private provider = 1 |
| Given ORS | No ORS = 0, Given ORS = 1 |
| Given zinc | No zinc = 0, Given zinc = 1 |
| Very poor | Any other wealth quintile = 0, Second wealth quintile = 1 |
| Poor | Any other wealth quintile = 0, Third wealth quintile = 1 |
| Less poor | Any other wealth quintile = 0, Fourth wealth quintile = 1 |
| Least poor | Any other wealth quintile = 0, Fifth wealth quintile = 1 |
