# Supplementary material for: Protocol for the economic evaluation of the diarrhea alleviation through zinc and oral rehydration salt therapy at scale through private and public providers in rural Gujarat and Uttar Pradesh, India
Source: Implement Sci. 2014 Nov 19;9:164. doi: 10.1186/s13012-014-0164-2 (PMC4335371; doi:10.1186/s13012-014-0164-2)
Supplement: Supplementary file 5 — Authors’ original file for figure 5 [file 13012_2014_164_MOESM5_ESM.docx]

**Table 4.** Descriptive statistics about costs

| **Costs according to source of care** |
| --- |
| **Public source - facility care** |
| PHC, government hospital, government dispensary |
| Auxiliary nurse midwife, subcenter |
| **Public source - community care** |
| Anganwadi worker/center |
| ASHA |
| **Private source** |
| Private doctor |
| Nursing home/private hospital |
| Mobile clinic |
| Chemist |
| Traditional healer |
| Charitable hospital, NGO, Trust |
| **Cost components according to outpatient, inpatient, and home care** |
| **Direct medical** |
| Consultation |
| Dispensing |
| Purchase of zinc (tablets or syrup) |
| Purchase of ORS (packets) |
| Purchase of other drugs |
| Special food purchased |
| Other costs |
| **Direct nonmedical** |
| Transportation (round trip) |
| **Indirect costs** |
| Wages lost |
